# Targeting the cholinergic anti-inflammatory pathway with vagus nerve stimulation in patients with Covid-19?

**DOI:** 10.1186/s42234-020-00051-7

**Published:** 2020-07-29

**Authors:** Bruno Bonaz, Valérie Sinniger, Sonia Pellissier

**Affiliations:** 1 Division of Hepato-Gastroenterology, Grenoble University Hospital, 38000 Grenoble, France; 2 Univ. Grenoble Alpes, Inserm, U1216, Grenoble Institute Neurosciences, 38000 Grenoble, France; 3 Univ. Grenoble Alpes, Univ. Savoie Mont Blanc and LIP/PC2S, 38000 Grenoble, France

**Keywords:** Cholinergic anti-inflammatory pathway, COVID-19, SARS-CoV-2, Vagus nerve, Vagus nerve stimulation, α7 nicotinic acetylcholine receptor

## Abstract

Severe acute respiratory syndrome coronavirus 2 (SARS-CoV-2), at the origin of the worldwide COVID-19 pandemic, is characterized by a dramatic cytokine storm in some critical patients with COVID-19. This storm is due to the release of high levels of pro-inflammatory cytokines such as interleukin (IL)-1 β, IL-6, tumor necrosis factor (TNF), and chemokines by respiratory epithelial and dendritic cells, and macrophages. We hypothesize that this cytokine storm and the worsening of patients’ health status can be dampened or even prevented by specifically targeting the vagal-driven cholinergic anti-inflammatory pathway (CAP). The CAP is a concept that involves an anti-inflammatory effect of vagal efferents by the release of acetylcholine (ACh). Nicotinic acetylcholine receptor alpha7 subunit (α7nAChRs) is required for ACh inhibition of macrophage-TNF release and cytokine modulation. Hence, targeting the α7nAChRs through vagus nerve stimulation (VNS) could be of interest in the management of patients with SARS-CoV-2 infection. Indeed, through the wide innervation of the organism by the vagus nerve, especially the lungs and gastrointestinal tract, VNS appears as a serious candidate for a few side effect treatment that could dampen or prevent the cytokine storm observed in COVID-19 patients with severe symptoms. Finally, a continuous vagal tone monitoring in patients with COVID-19 could be used as a predictive marker of COVID-19 illness course but also as a predictive marker of response to COVID-19 treatment such as VNS or others.

## Introduction

Severe acute respiratory syndrome coronavirus 2 (SARS-CoV-2) first appeared in December 2019, in Wuhan, Hubei Province of China, and rapidly spread in a worldwide pandemic. The vast majority of patients with the coronavirus disease 2019 (COVID-19) have a good prognosis. Clinically, SARS-CoV-2 ranges from asymptomatic disease to mild symptoms such as fever, sore throat, cough, loss of smell and taste, myalgia, headache, fatigue, to severe pneumonia with respiratory failure, acute kidney and/or cardiac injury, and death (Huang et al. 2020). Digestive manifestations such as nausea, vomiting, diarrhea, are also reported (Pan et al. 2020). Transmission of the virus occurs mainly through respiratory droplets. SARSCoV-2 RNA has been isolated in nasopharyngeal swabs, sputum, and in stool samples (Wang et al. 2020). This virus can attack lung cells by binding to the Angiotensin Converting Enzyme-2 (ACE2) receptor, and its presence in host cells will initiate various defensive responses leading to pneumonia and acute respiratory distress syndrome. ACE2 is also present in enterocytes of the small intestine thus explaining the digestive manifestations (Zhang et al. 2020). The complex SARS spike protein-ACE2 is proteolytically processed by the transmembrane serine protease 2 leading to cleavage of ACE2 and activation of the spike protein, which facilitates viral entry into the target cell (Hoffmann et al. 2020). Aging, male gender, obesity, diabetes, cardiovascular, kidney, and respiratory diseases are major aggravating factors for SARS-CoV-2 infection.

Proinflammatory responses play a role in the pathogenesis of human coronavirus. In vitro cell experiments have shown that delayed release of cytokines and chemokines occurs in respiratory epithelial cells, dendritic cells, and macrophages at the early stage of SARS-CoV infection. Later, the cells secrete low levels of the antiviral factors interferons and high levels of proinflammatory cytokines (IL-1 β, IL-6, and TNF) and chemokines (CCL-2, CCL-3, and CCL-5) (Ye et al. 2020). Clinical studies have detected such a cytokine storm in critical patients with COVID-19 which can result in acute lung injury and further progress to acute respiratory distress syndrome (Ye et al. 2020). Thus, controlling or preventing the inflammatory response may be an effective way of preventing collateral damage caused by the excessive activation of the immune system to clear pathogens.

### The cholinergic anti-inflammatory pathway and the vagus nerve

In 2000, the Tracey group introduced the concept of the cholinergic anti-inflammatory pathway (CAP). Indeed, a septic shock-induced increase of TNF in the liver and the blood in mice was dampened by stimulation of the distal end cut of the vagus nerve thus arguing for an anti-inflammatory effect of vagal efferents which release acetylcholine (ACh) (Borovikova et al. 2000). This group showed that nicotinic ACh receptor alpha7 subunit (α7nAChRs) is required for ACh inhibition of macrophage TNF release (Wang et al. 2003). Besides TNF, other pro-inflammatory cytokines such as IL6, IL1β was significantly decreased by vagus nerve stimulation (VNS) but not the anti-inflammatory cytokine IL-10. Consequently, we hypothesize that targeting the CAP with VNS could be of interest in patients with COVID-19. To reinforce this hypothesis, Staats et al. (Staats et al. 2020) have recently reported the clinical benefit of VNS in two patients with respiratory symptoms similar to those associated with COVID-19 but these preliminary results need to be confirmed in a larger randomized double-blinded control study.

**Fig. 1:**
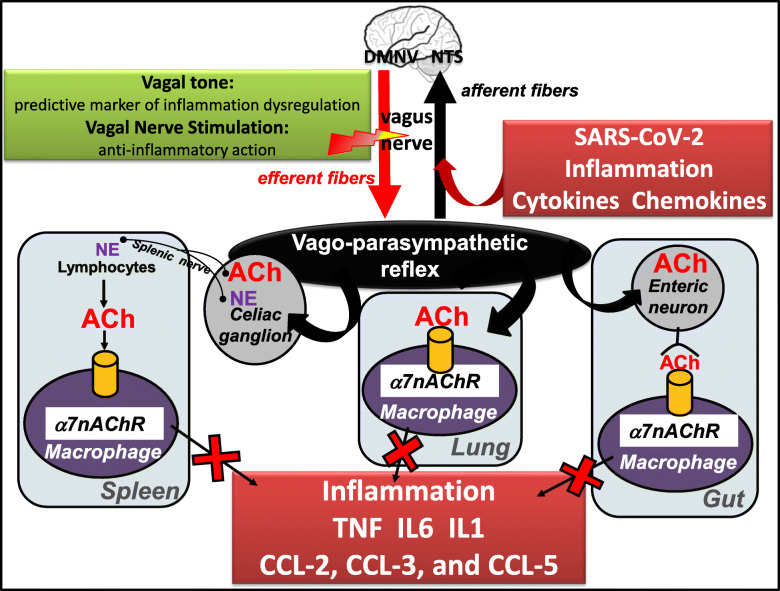
Vagus nerve stimulation: an anti-inflammatory tool targeting nAChR at multilevel organs in COVID-19. DMNV, dorsal motor nucleus of the vagus; NE, norepinephrine; NTS, nucleus tractus solitarius

The vagus nerve, the longest nerve of the organism, innervates the lungs and the gastrointestinal (GI) tract, two organs which are targeted by COVID-19. ACh released at the distal end of the vagus nerve acts on intrinsic neurons of the enteric nervous system e.g. at the level of the GI tract to inhibit the release of TNF by macrophages (Matteoli et al. 2014) (Fig. 1). A vago-splenic pathway inhibiting the release of TNF by splenic macrophages has also been described by the Tracey group  (Fig. 1). This effect is mediated through the link of norepinephrine on β2-adrenergic receptors of splenic CD4+ T cells that release ACh acting on α7nAChRs of splenic macrophages (Rosas-Ballina et al. 2008) i.e. the non-neuronal cholinergic pathway. The intra-cellular signaling of α7nAChRs inhibits transactivational activity of the transcription factor NF-kB p65 (Wang et al. 2004) and activates Jak2 and STAT3 signaling (de Jonge et al. 2005). Thus, targeting α7nAChRs with VNS should be of interest in the management of patients with COVID-19.

### Smoking and nicotine in COVID-19

The detrimental effect of smoking on cardiovascular and pulmonary disease has long been recognised. Thus, smoking should be an aggravating factor of COVID-19 infection. However, data are contradictory since if for some, smoking is associated with a negative progression and adverse outcomes (Alqahtani et al. 2020), for others it has a protective role (Farsalinos et al. 2020a). The protective effect of smoking is well known in ulcerative colitis, a chronic inflammatory bowel disease (IBD) (Berkowitz et al. 2018). Recently, Miyara et al. (Miyara et al. 2020) looked more closely at 482 patients positive for COVID-19 at the Pitié-Salpêtrière hospital in Paris and found that among the 343 patients hospitalised for serious complications and the 139 patients who were sent home, 4.4 and 5.3% were daily smokers respectively while the rate of daily smokers is 25.4% in the country. They suggest that smoking protection is likely mediated by nicotine, which alters the homeostasis of the renin-angiotensin system. They conclude that nicotine administration may be tested as a therapy to recapitulate the protecting effect of smoking against SARS-CoV-2 infection. Obviously, smoking cannot be recommended as a way of protecting oneself against the new coronavirus.

An increased ACE2 expression in airways of current smokers has been recently described by Leung et al. (Leung et al. 2020) and Russo et al. (Russo et al. 2020) with important implications for COVID-19 patients. These authors showed that nicotine upregulates ACE2 through α7nAChRs which are present in neuronal and non-neuronal cells. There is currently no evidence suggesting that up-regulation of ACE2 is associated with increased COVID-19 susceptibility or severity. In fact, up-regulation of ACE2 appears to be protective against tissue damage caused by SARS-CoV-2 (see for review Farsalinos et al.) (Farsalinos et al. 2020b). Indeed, ACE2 protects mice from developing acute respiratory distress syndrome (Imai et al. 2005). Continuous SARS-CoV-2 infection and replication induces down-regulation of ACE2, which may be implicated in organ damage and disease severity (Vaduganathan et al. 2020).

Recently, a hypothesis that the nicotinic cholinergic system may be involved in COVID-19 infection was presented, based on the fact that several of the symptoms and clinical signs of COVID-19, including the cytokine storm, could be explained by dysfunction of the CAP (Farsalinos et al. 2020b). Dysfunction of the nicotinic cholinergic system could be implicated in the thrombotic and vascular complications of COVID-19 (Farsalinos et al. 2020b).

Nicotine could provide protection through a direct action on various nAChRs expressed in neurons, immune cells, macrophages, cardiac tissue, lungs, and blood vessels (Kawashima et al. 2007). Changeux et al. (Changeux et al. 2020) hypothesize that the nAChR plays a key role in the pathophysiology of Covid-19 infection and might represent a target for the prevention and control of Covid-19 infection. There is a structural evidence supporting the hypothesis that SARS-Cov-2 virus is a nAChR blocker (Changeux et al. 2020). Consequently, α7nAChRs could be a candidate since they are expressed on immune cells regulating antigen-specific antibody and proinflammatory cytokine production and likely regulate the intensity of immune responses (Mashimo et al. 2020). Numerous immune cells, including T and B cells, macrophages, and dendritic cells, express the cholinergic system (Fujii et al. 2017). These immune cells belong to the non-neuronal cholinergic system and play a key role in regulating immune function using ACh acting on their own AChRs through autocrine and paracrine pathways. Among these AChRs, α7nAChRs play a role in the regulation of inflammatory responses and immune function as reported by the Tracey group.

### Vagus nerve stimulation in COVID-19

Thus, targeting α7nAChR activity could be of interest to modify immune function, which is dysregulated in COVID-19 infection where an immune system over-reaction or cytokine storm is observed (Fig. 1). In particular, targeting the CAP, a mechanism by which local inflammation activates the afferent (sensory) fibres of the vagus nerve to signal the brain to trigger an anti-inflammatory response through firing of the efferent vagus nerve i.e. the inflammatory reflex is of interest (Tracey 2002). Among the tools, VNS either invasive at the cervical level, validated in the treatment of drug resistant epilepsy and depression, or non-invasive through transcutaneous auricular or cervical stimulation could be of interest. VNS is a non-drug therapy able to stimulate the CAP thus dampening the release of pro-inflammatory cytokines such as TNF, IL6, IL1β. These pro-inflammatory cytokines are also involved in the pathogeny of chronic inflammatory disease such as IBD and rheumatoid arthritis (RA). VNS has recently shown its anti-inflammatory effect in IBD (Bonaz et al. 2016; Sinniger et al. 2020) and RA (Koopman et al. 2016a) especially by decreasing the plasmatic level of these cytokines. Consequently, VNS could be of interest in the management of patients with SARS-CoV-2 infection based on its modulatory effect on cytokine release. Indeed, through the wide innervation of the organism by the vagus nerve, especially the lungs and GI tract, VNS is a candidate as a treatment with few side effects as reported in epilepsy (Boon et al. 2018). Indeed, through the activation of α7nAChRs of macrophages by VNS at the level of the digestive system, the lungs and the spleen, VNS could dampen the cytokine storm observed in patients with severe Symptom*s. vagus* nerve fibers are present in the human lung, especially in the alveoli (Fox et al. 1980). Alveolar macrophages, epithelial cells and inflammatory infiltrated neutrophils express α7nAChR and could be the players at efferent arm of pulmonary parasympathetic inflammatory reflex (Su et al. 2010). The vagus nerve plays an important role in pulmonary inflammation (dos Santos et al. 2011). The lung tissue expresses the cholinergic system including nAChRs involved in the pulmonary parasympathetic inflammatory reflex (Yang et al. 2014). VNS is capable to regulate disequilibrium of the autonomic nervous system (high sympathetic nervous activity and low parasympathetic nervous activity) in an experimental model of acute lung injury (Liu et al. 2017) and acts through the CAP, by means of α7nAChR to prevent lung injury (Tarras et al. 2013). VNS alleviated lung injury through the reduction of gut and lung permeability through nAChR. Regarding the choice of stimulation parameters, those classically used in epilepsy could be of interest. In particular, a high frequency stimulation of 20 to 30 Hz, used for epilepsy, is known to target vagal afferents, which represent 80% of the vagus nerve fibers (Prechtl and Powley 1990). These vagal afferents target the central nervous system (CNS) through the nucleus tractus solitarius then activating the central autonomic network (Benarroch 1993), which modulates the autonomic nervous system, ie the sympathetic and parasympathetic nervous systems. The other possibility would be to use low-frequency stimulation of 5–10 Hz known to stimulate vagal efferents, and thus the CAP, although vagal afferents are also activated with such frequency (Reyt et al. 2010). In fact, activating both vagal afferent and efferent fibers is of interest to activate the CAP (Bonaz et al. 2019). If the optimal VNS parameters for resolution of inflammation are still unknown, Tsaava et al. (Tsaava et al. 2020) reported recently that specific combinations of pulse width, pulse amplitude, and frequency produced a significant increase of TNF, while other parameters selectively lowered serum TNF levels, as compared to sham-stimulated mice. They also showed that serum levels of IL-10 were significantly increased by select parameters of neurostimulation but were unchanged with others.

Based on the anti-inflammatory effect of VNS in chronic inflammatory disorders of the GI tract, VNS could have an effect on digestive manifestations due to the virus. Indeed, SARS-CoV-2 infection in patients with COVID-19 induces an inflammatory response in the gut, as evidenced by diarrhoea, elevated fecal calprotectin (expressed by neutrophil granulocytes), and a systemic IL-6 response (Effenberger et al. 2020). It is currently unknown if SARS-CoV-2 infection affects the course of IBD patients and whether immunosuppressive treatment affects their susceptibility to (or the course of) COVID-19, but the baseline use of biologics is not associated with worse COVID-19 outcomes in IBD patients (Haberman et al. 2020). However, active IBD, older age and presence of comorbidities are associated with a higher risk of COVID-19 pneumonia and death in patients with IBD (Bezzio et al. 2020).

The central effect of VNS could have also an interest in the neurological manifestations of COVID-19 which were observed in ~ 36.4% in a case series of 214 patients (Mao et al. 2020). Indeed, the intense systemic inflammation triggered by SARS-CoV-2 infection may lead to blood-brain barrier breakdown thus allowing peripheral cytokines to access to the CNS where they may trigger or exacerbate neuroinflammation, as reported in experimental model of postinfectious autoimmune encephalitis (Platt et al. 2020) that could contribute to neuroinflammatory processes and increase susceptibility to neurological syndromes. In addition, olfactory tract becomes an important channel for virus transmission to brain (Mori et al. 2005) and anosmia reported in patients COVID-19 is possibly connected to the intranasal route of infection. Coronavirus can invade the CNS from the periphery through neural pathways (Wu et al. 2020). In addition, the potential invasion to CNS of SARS-CoV-2 may be one reason for the acute respiratory failure. Indeed, some coronaviruses are able to spread via a synapse-connected route to the medullary cardiorespiratory centre from the mechanoreceptors and chemoreceptors in the lung and lower respiratory airways (Li et al. 2020). ACE2 is expressed in the nucleus tractus solitarius and dorsal motor nucleus of the vagus nerve (Doobay et al. 2007). Thus, the virus might infect the terminal areas of vagal afferents or the origin of vagal efferents inducing down-regulation of ACE2 and favouring local inflammation that could disrupt the CAP and dysregulate the inflammatory response.

Targeting the CNS with VNS could have a protective effect through a central anti-inflammatory effect. Indeed, VNS significantly reduced the central levels of pro-inflammatory cytokines and the percentage of microglia and macrophages in lipopolysaccharide stimulated mice (Meneses et al. 2016). Targeting plasticity with VNS to renormalize aberrant neural circuits and restore normal function is also of interest (Hays et al. 2013). A vagus nerve-neurotrophin interaction model in the brain has been recently proposed supporting a putative role of Nerve Growth Factor and Brain Derived Neurotrophic Factor in the mechanism of action of VNS to modulate brain activities (Rosso et al. 2020).

### Monitoring vagal tone in COVID-19

Vagal tone can be explored indirectly and through a non-invasive measure namely heart rate variability. Variations in the duration of successive RR intervals are dependent on the efferent output of the sympathetic and parasympathetic nerves. Under resting condition, this measure reflects mainly the vagal tone on heart rhythm (Heart rate variability: standards of measurement, physiological interpretation and clinical use 1996). Vagal tone is dampened in stress condition either of inflammatory and/or infectious origin. The level of resting vagal tone may give some indications about the individual vulnerability face to the stressors (i.e. the virus or inflammation). In particular, we have shown that in patients with Crohn’s disease in remission, there is an inverse relation between vagal tone and TNF in the blood (Pellissier et al. 2014) and that VNS is able to restore vagal tone (Bonaz et al. 2016; Sinniger et al. 2020). A lower parasympathetic activity and a decreased expression level of α7nAChR on peripheral blood monocytes, and higher sympathetic hormone (norepinephrine) is observed in patients at risk to develop RA (Koopman et al. 2016b). Thus, a dysautonomia precedes the development of RA. An autonomic dysfunction is reported in various pathological conditions such as obesity, diabetes, hypertension, pulmonary diseases (Zalewski et al. 2018), which are risk factors for aggravating COVID-19 infection. Monitoring vagal tone in patients with COVID-19 could be of two major interests. Firstly, it can be a predictive marker of response to COVID treatment such as VNS. Secondly, it could be used as a predictive marker of COVID illness course with the idea that people with very low vagal tone at the onset of the symptoms may be at high risk to develop a dysregulated overstimulated pro-inflammatory response during the infection leading to sudden death or intensive care unit transfer. These patients could thus benefit from VNS. Consequently, both targeting α7nAChR with VNS and an early continuous monitoring of vagal tone could be of interest in the management of patients with COVID-19 infection. VNS is a slow-acting therapy, as demonstrated in epilepsy (Elliott et al. 2011) and as we have observed in patients with Crohn’s disease since the delay of onset of action was ~ 3 months (Bonaz et al. 2016; Sinniger et al. 2020). Consequently, VNS could be more appropriate in moderate forms of the disease and in the prevention of the cytokine storm in patients with low vagal tone as indexed by careful standard measures of heart rate variability. However, SARS-CoV-2 is a more acute inflammatory condition, especially the cytokine storm, than Crohn’s disease which is a chronic inflammatory condition. Thus, one can think that VNS should be more rapidly active as observed in acute inflammation in models of lipopolysaccharide-induced TNF serum levels or postoperative ileus (Stakenborg et al. 2017). In addition, VNS could accelerate the post-COVID recovery and further VNS could reduce the posttraumatic stress syndrome classically described in such patients (Xiao et al. 2020).

## Conclusion

Based on the anti-inflammatory effects of VNS both in the periphery and in the CNS, VNS and continuous monitoring of vagal tone through heart rate variability appears as two particularly innovative applications in COVID-19 patient management.

## Data Availability

NA

## Abbreviations

ACE2: Angiotensin converting enzyme-2 receptor
ACh: Acetylcholine
CAP: Cholinergic anti-inflammatory pathway
CCL: Chemokine ligands
CNS: Central nervous system
COVID-19: Coronavirus disease 2019
GI: Gastrointestinal
IBD: Inflammatory bowel disease
IL: Interleukin
RA: Rheumatoid arthritis
SARS-CoV-2: Severe acute respiratory syndrome coronavirus 2
TNF: Tumor necrosis
VNS: Vagus nerve stimulation
α7nAChRs: α7 nicotinic acetylcholine receptor
